# Management of postintubation tracheal stenosis in a neurosurgical patient with tracheomalacia and scarring tendency: A case report

**DOI:** 10.1097/MD.0000000000048392

**Published:** 2026-04-17

**Authors:** Yewen Zhan, Shurong Zhang, Min Chen, Hong Pu

**Affiliations:** a Department of Critical Care Medicine, West China School of Medicine, West China Hospital of Sichuan University, Chengdu, Sichuan Province, PR China; b Department of Otolaryngology, West China School of Medicine, West China Hospital of Sichuan University, Chengdu, Sichuan Province, PR China.

**Keywords:** airway management, case report, laryngotracheal stenosis, neurosurgery, postintubation tracheal stenosis, scarring tendency, tracheomalacia

## Abstract

**Rationale:**

Postintubation tracheal stenosis, a rare and life-threatening iatrogenic complication arising from the endotracheal intubation. This case is noteworthy for a very young adult with a neurosurgical background and clustered risk factors, who developed rapidly progressive high-grade subglottic/cervical tracheal stenosis with concomitant tracheomalacia.

**Patient concerns:**

This report details a 19-year-old Han Chinese man, who is a university student, developed severe dyspnea after surgery of bilateral ventricular drainage due to cerebral hemorrhage 48 days ago. His intensive care unit course included 10 days of mechanical ventilation, gastric reflux, and bloodstream and airway infections. After first discharge, he developed recurrent, escalating inspiratory dyspnea (repeatedly misdiagnosed as pneumonia or asthma).

**Diagnoses:**

Lack of response to bronchodilators and steroids, absence of diffuse lower-airway process on imaging, and direct visualization of a fixed, cicatricial, high-grade subglottic stenosis on bronchoscopy together argued against alternative diagnoses including asthma, vocal cord dysfunction, and recurrent pneumonia. Grade 3 subglottic tracheal stenosis that the 6 mm bronchoscope could not pass through was confirmed by three-dimensional reconstruction computed tomography. A diagnosis of upper tracheal obstruction caused by postintubation tracheal stenosis was considered.

**Interventions:**

Multidisciplinary consensus favored emergency laryngotracheal stenosis resection, laryngeal function reconstruction, and tracheoplasty. Successful extubation was achieved on the first postoperative day and discharged on postoperative day 10.

**Outcomes:**

Follow-up imaging confirmed sustained airway patency without restenosis. He achieved complete symptomatic relief, then promptly returned to the university and successfully graduated 2 years later. Until now, he has been employed full-time without functional limitations.

**Lessons:**

In young patients with high-grade, subglottic, cicatricial stenosis and tracheomalacia, primary resection with anastomosis can be definitive when endoscopic or stent options are unsuitable in an emergency. Early warning signals (reflux, infection, post-extubation cough/stridor, and poor response to pharmacotherapy) warrant timely evaluation. Durable outcomes depend on an experienced multidisciplinary team cooperation and standardization of diagnostic, therapeutic, and follow-up pathways.

## 1. Introduction

Mechanical ventilation (MV) serves as a cornerstone of respiratory support for critically ill patients, with studies indicating that approximately 72% of patients admitted to the intensive care unit (ICU) require MV.^[1]^ Postintubation tracheal stenosis (PITS) arising from endotracheal tube (ETT) cuff pressure is a rare yet severe complication predominantly observed in patients undergoing prolonged MV and poses a persistent clinical challenge owing to its risk of misdiagnosis, recurrence, and complex treatment.^[2]^ The incidence of PITS ranges from 0.6% to 21%, as reported.^[3]^ Despite advancements in surgical techniques and endotracheal device design aimed at mitigating iatrogenic complications, PITS remain life-threatening for patients requiring ventilation assistance due to critical stenosis.^[4]^ It also places a heavy burden on patients and the health care system.^[5]^ The pandemic of coronavirus disease 2019 has exacerbated this challenge, driving a global surge in long-term MV utilization.^[6]^ It is expected that more PITS will be detected in patients with prolonged MV. Despite its diagnostic challenges and frequent need for emergent interventions, standardized guidelines for PITS management are absent.

This study describes a case of PITS with tracheomalacia and scarring tendency secondary to prolonged oral intubation that was successfully treated by emergency laryngotracheal stenosis resection, laryngeal function reconstruction, and tracheoplasty in a tertiary teaching and academic hospital. Although PITS has been reported across age groups, occurrence in a very young adult with a recent neurosurgical history and a scarring-prone phenotype presents a distinctive clinical scenario. To our knowledge, the concurrence of these risk factors in a single emergent presentation is rare, and it underscores specific diagnostic and perioperative challenges that may inform best practices for similar cases.

## 2. Case presentation

A 19-year-old Han Chinese man, who was a university student, was admitted to the ICU following recurrent dyspnea, wheezing, and inspiratory stridor persisting for 5 hours (see Table 1 for a clear timeline of clinical key events). He underwent bilateral ventricular drainage due to cerebral hemorrhage 48 days ago, followed by 10 days of mechanical ventilation. During the first oral intubation, gastric reflux occurred, necessitating jejunal tube placement. Subsequent sepsis episodes (during intubation and post-extubation) were attributed to *Streptococcus anginosus* in the blood, *Pseudomonas aeruginosa*, and *Staphylococcus aureus* in the alveolar lavage fluid, as suggested by metagenomic next-generation sequencing. The first post-extubation fiberoptic bronchoscopy revealed tracheal-wall erosion with extensive necrotic tissue in the subglottis and upper tracheal segment. Thereafter, the patient gradually developed a dry cough that was independent of day–night rhythm and increasingly disturbed his sleep. In line with these findings and the chest-computed tomography (CT) images, we initiated antibiotics, acetylcysteine, and ambroxol hydrochloride nebulization. Follow-up tests showed a drop in procalcitonin and C-reactive protein and radiographic resolution of pneumonia, yet the dry cough persisted. A traditional Chinese medicine consultation was then sought; after herbal decoction, the cough improved and the patient was discharged from our hospital.

**Table 1:** Timeline of clinical key events.

Time	Event
Day 1	Endotracheal intubation and neurosurgical drainage surgery
Days 2 to 10	Mechanical ventilation; reflux; sepsis episodes; extubation
Days 11 to 39	Dry cough developed gradually; bronchoscopy: subglottic erosion/necrosis
Day 40	First discharge
Day 48	Readmission with severe inspiratory dyspnea; CT 3D and bronchoscopy: grade 3 subglottic/cervical stenosis
Day 49 (surgery)	Emergency laryngotracheal stenosis resection, laryngeal function reconstruction, and tracheoplasty
POD 1	Bronchoscopy and leak test satisfactory; extubation per objective criteria
POD 10	Second discharge
POD 50	Asymptomatic; CT: no restenosis or leak
Post-op 2 years	Graduated and employed full-time (durable functional recovery)

CT = computed tomography, POD = postoperative day, post-op = postoperative.

Eight days after his initial discharge, the patient presented to the emergency department with recurrent dyspnea characterized by pronounced shortness of breath and marked inspiratory dyspnea. Provisional diagnoses of pneumonia and bronchial asthma prompted the immediate administration of bronchodilators and broad-spectrum antibiotics. Despite these interventions, his respiratory distress persisted, with deteriorating oxygen saturation levels necessitating escalated care. Consequently, he was admitted to the ICU for advanced respiratory support via noninvasive ventilation to stabilize gas exchange and mitigate hypoxemic compromise.

**Figure 1.**
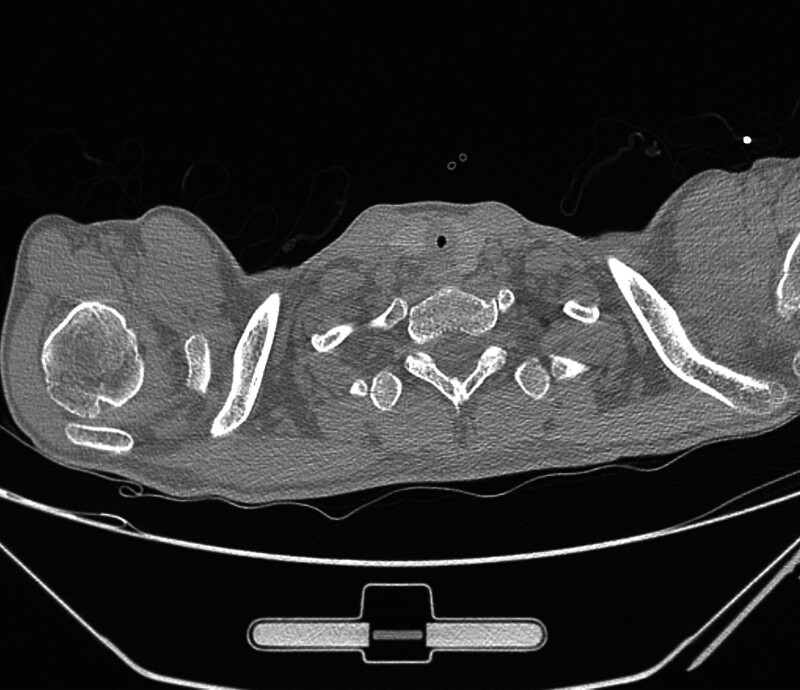
CT image of stenotic trachea at the second admission. CT = computed tomography.

A non-contrast CT scan with three-dimensional reconstruction demonstrated cervical tracheal stenosis (TS) (Fig. 1), further confirmed by bronchoscopy, demonstrating normal vocal cord mobility and Grade 3 subglottic tracheal stenosis (a 6 mm bronchoscope was unable to pass through the lesion). The scarring tendency of the patient was provided by his parents after bronchoscopy. His dyspnea gradually progressed, corticosteroids were ineffective, and his oxygenation could not be maintained by noninvasive ventilation. After multidisciplinary consultation, a diagnosis of upper tracheal obstruction caused by PITS was considered. Before confirming fixed tracheal stenosis, alternative diagnoses included asthma, vocal cord dysfunction, and recurrent pneumonia. Lack of response to bronchodilators and steroids, absence of diffuse lower-airway process on imaging, and direct visualization of a fixed, cicatricial, high-grade subglottic stenosis on bronchoscopy together argued against these alternatives.

Multidisciplinary discussion considered but rejected emergent stenting because of the high subglottic location with cricoid involvement, circumferential cicatricial narrowing, and coexistent tracheomalacia (anatomical features associated with poor stent stability and higher risks of migration and granulation). In addition, urgent availability of an appropriately sized silicone stent could not be guaranteed in the emergency timeframe, and metallic stents are discouraged in benign disease. Consequently, emergency laryngotracheal stenosis resection, laryngeal function reconstruction, and tracheoplasty were performed under general anesthesia.

**Figure 2.**
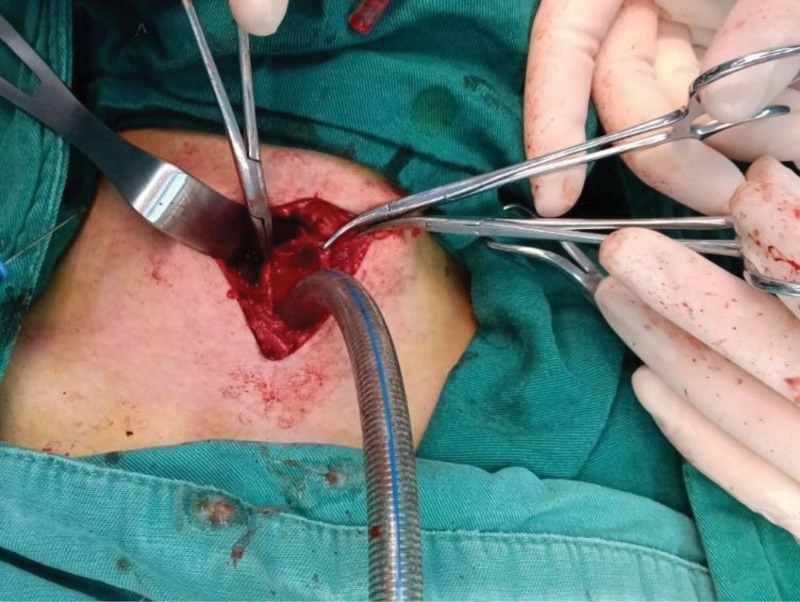
Ventilation through the distal trachea maintained by an endotracheal tube.

**Figure 3.**
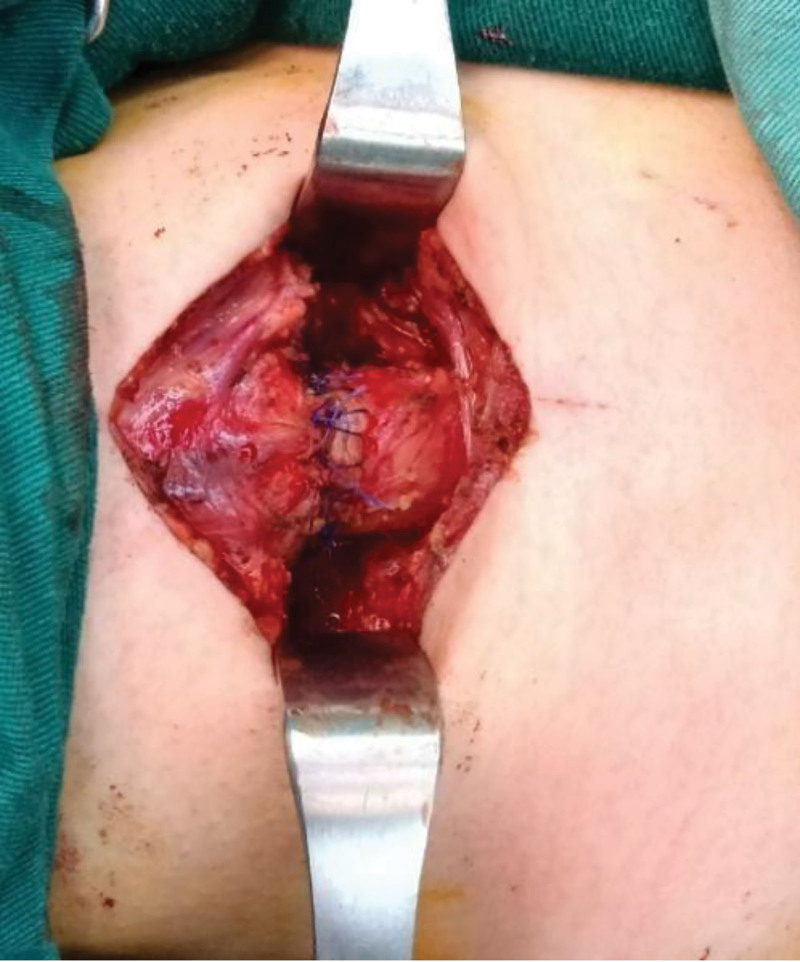
Anastomotic trachea after resection of stenotic segment.

The patient was placed in the supine position following local infiltration anesthesia in front of the neck. The skin and subcutaneous tissue were cut vertically with a 5 cm longitudinal incision along the front of the neck. After exposing a part of the cervical trachea, a raised scar was observed in front of the trachea. Additionally, tracheomalacia of the 1st to 5th tracheal cartilage rings was noted at the inferior edge of the cricoid cartilage, with only a small fissure in the tracheal wall. Circumferential resection was performed at the level of the stenosis and 2 to 3 mm normal mucosal margins of the resection. Following transection below the lesion and placement of lateral stay sutures, MV through the distal trachea was maintained using an ETT, while the stenosis from the 1st to 5th tracheal cartilage rings was resected (Fig. 2). After resection, the posterior wall of the mobilized cervical trachea was anastomosed end-to-end to the inferior edge of the cricoid cartilage with interrupted 4-0 vicryl absorbable sutures under no tension, facilitated by prior tracheal release. An oral ETT was then advanced beyond the anastomosis and completed in a similarly interrupted fashion (Fig. 3). At the end of the surgery, the patient’s chin was sutured to the presternal skin to relieve the postoperative hyperextension of the anastomosed trachea with 2-0 nylon sutures. The chin-to-presternal sutures reduced anastomotic tension via 30° to 45° neck flexion to prevent dehiscence, maintained alignment by avoiding neck hyperextension, promoted mucosal apposition and accelerated healing.^[7,8]^ Anesthetic management prioritized oxygenation and airway security. Cross-field endotracheal intubation with intermittent cross-field ventilation was employed during tracheal transection and resection, coordinated with periods of oral endotracheal ventilation by the anesthesiologist. Continuous capnography and arterial blood gas monitoring were used to guide ventilation, and high-fidelity communication between surgical and anesthesia teams minimized apnea intervals.

**Figure 4.**
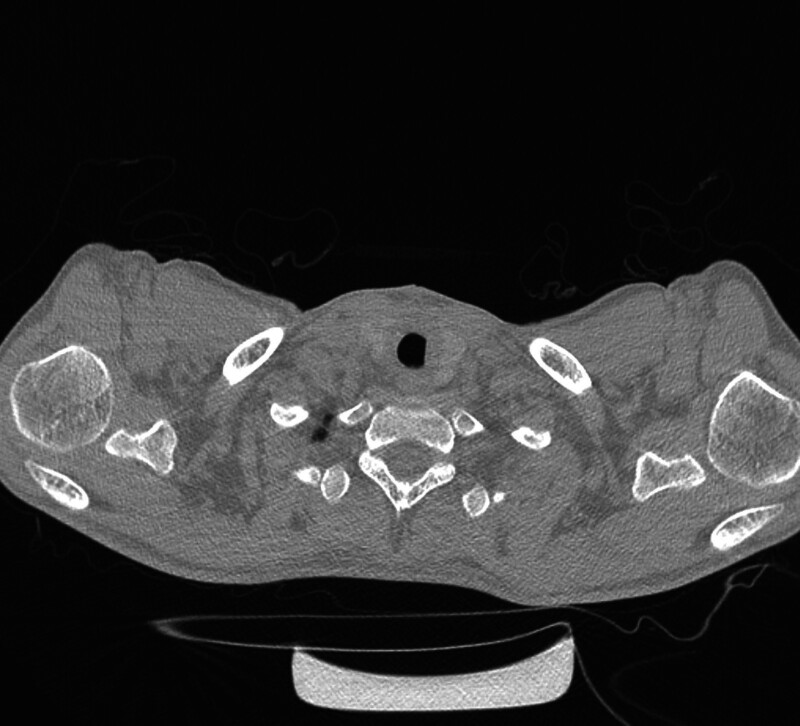
CT image of trachea on the 10th postoperative day. CT = computed tomography.

**Figure 5.**
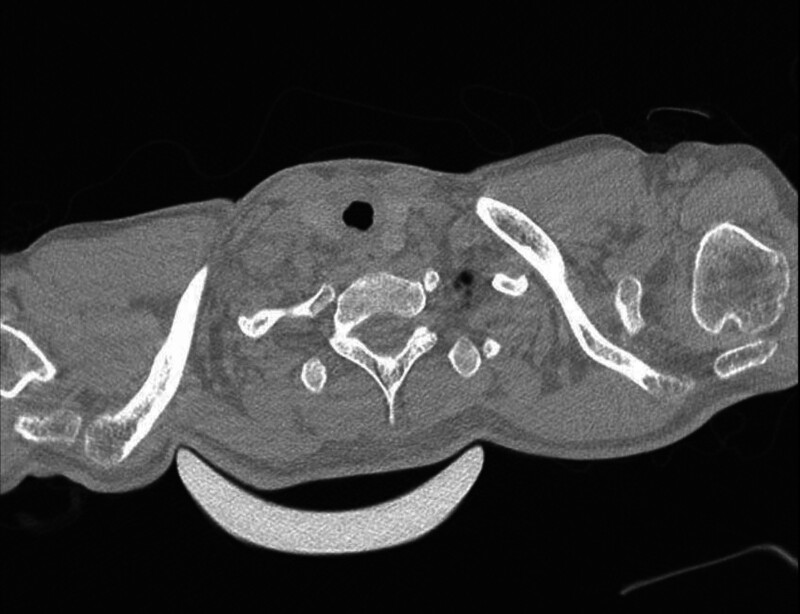
CT image of trachea on the 50th postoperative day. CT = computed tomography.

On the first postoperative day (POD), the patient was extubated in the ICU by the following extubation criteria: PaO_2_/FiO_2_ >300 mm Hg, tidal volume >5 mL/kg, negative inspiratory force <‐25 cm H_2_O, and bronchoscopy-confirmed airway patency.^[9]^ Contingency plans included a standby 6.0 mm ETT, bronchoscope, tracheostomy kit, and 24-hour ICU observation with continuous pulse oximetry.^[10]^ On the POD 10, CT showed restoration of the tracheal lumen with unequivocal patency (Fig. 4). Beyond mild transient cervical discomfort, no complications occurred (no pressure ulcers, dysphagia, neck/shoulder pain, anastomotic leak, infection, or early restenosis). At 1-month follow-up, he reported the ability to ambulate without dyspnea and had no hypoxemia or stridor; clinical exam suggested a normal voice without dysphonia. On the POD 50, CT still revealed no restenosis or dehiscence (Fig. 5).

After being discharged from the hospital, he promptly returned to the university and successfully graduated 2 years later, in June 2025. Now he has started full-time employment without functional limitations. Afterwards, the young man recalled his experience: “Repeated ICU admissions with near-suffocation from tracheal stenosis left me in profound fear. Constant dyspnea and dry cough caused every breath to struggle. Antibiotics initially reduced inflammation; however, when severe dyspnea violently recurred, I hoped to despair. Emergency neck surgery, although daunting, is inevitable. Postoperatively, breathing effortlessly provided immediate relief. Discomfort from neck sutures and chin fixation was negligible compared to the restored airflow. During follow-up rehabilitation, I had no difficulties in my daily life. I returned to school on time, communicated normally with classmates and friends as usual, played basketball, and graduated smoothly to start working. Sometimes the suffocation experience feels like nothing more than a dream. Critical illness demands medicine and resilience.”

## 3. Discussion

This manuscript details the successful management of a rare case of severe PITS in a young patient with a neurosurgical history and multiple risk factors. The case was unique due to the patient’s age, the rapid progression of the stenosis, and the coexistence of high-grade subglottic cicatricial stenosis with associated tracheomalacia.

Laryngotracheal stenosis is considered the result of an aberrant wound-healing process that leads to fibrotic scarring, arising from a variety of etiologies. Iatrogenic causes, including PITS, are the primary causes of subglottic or tracheal stenosis.^[11]^ PITS accounted for 60.7% of iatrogenic TS, as reported in a study of 205 patients, and almost half of the PITS patients had more complex stenosis (mixed stenosis, 48.9%) than post-tracheostomy tracheal stenosis, which was also seriously life-threatening.^[12]^ PITS appears from days to decades after extubation. Only when the cross-sectional area of the trachea has decreased by at least 70% will they cause breathing difficulties in the resting state.^[13]^ The most common symptoms in patients with TS are shortness of breath, wheezing, coughing, and voice changes, which are often misdiagnosed as difficult-to-control asthma or chronic obstructive pulmonary disease and partially improved with the use of cortisol, bronchodilators, and antibiotics. Up to 44% of patients are not diagnosed when symptoms first appear, and a small number of patients may have a delayed diagnosis of up to 3 months after intubation.^[14]^ All of these characteristic features of PITS were exemplified in this patient’s course, making the case a high-yield teaching example. Prolonged 10-day mechanical ventilation, gastric reflux requiring jejunal tube placement, and tracheal mucosal erosion with necrosis represented early warning signs for PITS. Prolonged intubation (>7 days) is a well-established risk factor for ETT-induced stenosis,^[13]^ while reflux exacerbates mucosal damage,^[14]^ and triggers an aberrant wound-healing process that directly leads to cicatricial narrowing.^[11]^ The immediate cough after extubation, concurrent radiographic signs of pneumonia initially, and traditional Chinese medicine proved effective led us to a diagnosis of “simple pneumonia”; therefore, CT and fiber-optic bronchoscopy should be included in the diagnostic work-up to ensure accurate diagnosis.

Endoscopic interventions, including balloon dilation, ablation therapy, and stent implantation, constitute the first-line treatment for mild-to-moderate PITS.^[15]^ However, stent infeasibility in this patient was driven by 3 factors: youth: for young patients with decades of life expectancy, a stent is only a temporary bridge; long-term complication rates (infection, obstruction, fracture, and difficult extraction) are significantly higher than those of resection and reconstruction.^[16]^ Anatomical constraints (tracheomalacia of the 1st to 5th cartilage rings (intraoperatively confirmed) contraindicate stents, as they cannot support collapsed cartilage,^[9]^ and subglottic stenosis increases migration risk);^[15]^ urgency (impending respiratory failure required immediate airway restoration, and stent sizing/fluoroscopic placement would delay intervention).^[7]^

Surgical resection remains the definitive treatment for endoscopic intervention failure or severe TS (Cotton-Myer grades 3 and 4), that is, the cross-sectional area of the narrow part of the airway is >70%, or even completely blocked.^[9]^ Laryngotracheal reconstruction (LTR) and cicatricial stenosis resection with end-to-end anastomosis are the predominant surgical interventions for airway stenosis, as evidenced in prior studies.^[9]^ LTR is typically indicated for cases involving structurally preserved laryngotracheal cartilage frameworks, whereas resection-anastomosis techniques are reserved for severe subglottic or TS accompanied by extensive cricoid cartilage defects.^[8]^ LTR requires intact cartilage,^[9]^ while the patient had 1st to 5th ring malacia. Prolonged stenting with LTR will delay extubation.^[7,12]^ Studies have reported 90% 1-year patency for segmental resection (vs 75% for LTR) and no voice impairment.^[8]^ Alternative approaches, such as the Maddern or Reacher procedures, prioritize mucosal scar excision while preserving the tracheal cartilage integrity. These techniques demonstrate superior outcomes relative to conventional surgeries, including fewer iatrogenic complications and enhanced long-term patency, rendering them particularly advantageous for idiopathic subglottic stenosis management.^[17]^ Despite advancements, surgical decision-making remains influenced by institutional protocols and operator expertise.

Perioperative anesthesia management poses unique challenges in such cases, as the shared surgical-anesthetic airway demands meticulous coordination to ensure sustained oxygenation during the complex excisional or reconstructive phases.^[10]^ Oxygenation was maintained via distal tracheal intubation with a cuffed ETT, consistent with tracheal resection guidelines.^[18]^ Anesthetic challenges in shared airway were mitigated by real-time surgeon–anesthesiologist coordination to minimize apneic intervals,^[10]^ reasonable general anesthesia analgesics and sedatives to maintain hemodynamic stability and prevent anastomotic bleeding. Continuous capnography ensured adequate ventilation during ETT repositioning, preventing hypercapnia. Contemporary strategies emphasize modular ventilation systems that enable dynamic intraoperative adaptation through hybrid approaches. These may integrate intermittent apneic intervals, high-frequency jet ventilation, laryngeal mask airways, or preserved spontaneous respiration tailored to procedural requirements and anatomical constraints.^[18]^ Nevertheless, evidence regarding the outcome-related risks and benefits of novel airway management procedures is limited. Extracorporeal support can ensure adequate gas exchange without interfering with the airway and surgical field, and can often be used as an effective rescue technique in cases where other approaches have failed.^[7]^

Cicatractomy for ``laryngotracheal stenosis + laryngeal function reconstruction + tracheoplasty’’ was therefore prioritized as the most durable option. Distal tracheal intubation maintained operative oxygenation, and the interrupted suture successfully connected the distal and proximal trachea. Although topical mitomycin C or corticosteroid strategies have been described to mitigate granulation,^[15]^ evidence remains heterogeneous, and potential wound-healing effects warrant caution^[9]^ in the emergent setting. Given minimal granulation at surgery and a stable anastomosis, we elected not to use adjuncts, favoring meticulous technique and tension mitigation. Tension reduction was achieved by chin-to-presternal 2-0 nylon sutures^[7]^ and restenosis was prevented by sharp dissection (preserving mucosal perfusion) and residual scar inspection.^[9]^ Long-term validation is equally robust: asymptomatic air patency at 1 month, CT-confirmed absence of leak or restenosis at POD 50, and full social reintegration (graduation plus full-time employment) at 25 months (anatomical and functional durability that extends well beyond the usual short-term endoscopic reports). These outcomes underscore the value of ``segmental resection + laryngeal release + 7-day chin-to-chest fixation’’ in patients with challenging cicatricial tendencies.

This satisfactory outcome was attributable to coordinated multidisciplinary collaboration: emergency physicians expedited recognition and transfer; anesthesiologists secured a difficult airway and executed cross-field ventilation; thoracic and ENT surgeons jointly planned resection, performed resection of narrowed tracheal segment, and executed a tension-free anastomosis; and ICU teams standardized sedation weaning, extubation readiness assessment, and surveillance bronchoscopy. Such structuring may deliver a replicable model of best practice for managing emergency complex-airway scenarios. Therefore, a paradigm shift toward multidisciplinary collaboration is imperative for tailoring interventions to individual patient profiles. Furthermore, the current landscape underscores the urgent need for international consensus guidelines to standardize the diagnostic criteria and imaging/bronchoscopic reporting for PITS; timing of intervention in emergent versus elective settings; technical standards (release maneuvers, suture materials, and intraoperative endoscopic assessment) to reduce variability; and structured follow-up incorporating objective airway imaging and functional measures.

## 4. Conclusion

In conclusion, our findings reaffirm that resection with primary anastomosis remains the gold standard for treating severe PITS, particularly when complicated by cartilage injury or tracheomalacia and scarring tendency. Second, this case underscores that the successful outcome was contingent upon a standardized, multidisciplinary protocol, which is critical for the management of a shared-airway emergency. We also emphasize that early recognition of warning signs and timely intervention are essential to prevent catastrophic airway compromise. This systematic approach serves as a best-practice model and highlights the urgent need for international guidelines to standardize the management of this complex condition.

## Acknowledgments

We wish to thank the patient and all the researchers who participated in this study.

## Author contributions

**Conceptualization:** Yewen Zhan, Shurong Zhang, Min Chen, Hong Pu.

**Data curation:** Shurong Zhang.

**Validation:** Min Chen.

**Writing – original draft:** Yewen Zhan.

**Writing – review & editing:** Hong Pu.
